# Improved Personalised Neuroendocrine Tumours’ Diagnosis Predictive Power by New Receptor Somatostatin Image Processing Quantification

**DOI:** 10.3390/jpm11101042

**Published:** 2021-10-17

**Authors:** Cati Raluca Stolniceanu, Mihaela Moscalu, Doina Azoicai, Bogdan Tamba, Constantin Volovat, Irena Grierosu, Teodor Ionescu, Wael Jalloul, Vlad Ghizdovat, Roxana Gherasim, Simona Volovat, Feng Wang, Jingjing Fu, Roxana Moscalu, Milovan Matovic, Cipriana Stefanescu

**Affiliations:** 1 Department of Biophysics and Medical Physics-Nuclear Medicine, “Grigore T. Popa” University of Medicine and Pharmacy, 700115 Iasi, Romania; raluca.stolniceanu@umfiasi.ro (C.R.S.); irena.raileanu@umfiasi.ro (I.G.); teodor-marian-m-ionescu@d.umfiasi.ro (T.I.); jalloul.wael@umfiasi.ro (W.J.); vlad.ghizdovat@umfiasi.ro (V.G.); roxana-o-gherasim@d.umfiasi.ro (R.G.); cipriana.stefanescu@umfiasi.ro (C.S.); 2 Department of Preventive Medicine and Interdisciplinarity, “Grigore T. Popa” University of Medicine and Pharmacy, 700115 Iasi, Romania; 3 Department of Epidemiology, “Grigore T. Popa” University of Medicine and Pharmacy, 700115 Iasi, Romania; doina.azoicai@umfiasi.ro; 4 Advanced Center for Research and Development in Experimental Medicine (CEMEX), “Grigore T. Popa” University of Medicine and Pharmacy, 700454 Iasi, Romania; bogdan.tamba@umfiasi.ro; 5 Department of Medicine III—Medical Oncology-Radiotherapy, “Grigore T. Popa” University of Medicine and Pharmacy, 700115 Iasi, Romania; constantin.volovat@email.umfiasi.ro (C.V.); simona-ruxandra.volovat@umfiasi.ro (S.V.); 6 Department of Nuclear Medicine, Nanjing First Hospital, Nanjing Medical University, Nanjing 210006, China; fengwangcn@hotmail.com (F.W.); jingjingfu910730@hotmail.com (J.F.); 7 Manchester Academic Health Science Centre, School of Medical Sciences Manchester, The University of Manchester, Manchester M139PT, UK; roxana.moscalu@mft.nhs.uk; 8 Clinical Center Kragujevac, Center for Nuclear Medicine, Faculty of Medical Sciences, University of Kragujevac, 34000 Kragujevac, Serbia; mmatovic@medf.kg.ac.rs

**Keywords:** gamma emitters tracer, uptake indices, neuroendocrine tumours, NETs, quantification

## Abstract

Although neuroendocrine tumours (NETs) are intensively studied, their diagnosis and consequently personalised therapy management is still puzzling due to their tumoral heterogeneity. In their theragnosis algorithm, receptor somatostatin scintigraphy takes the central place, the diagnosis receptor somatostatin analogue (RSA) choice depending on laboratory experience and accessibility. However, in all cases, the results depend decisively on correct radiotracer tumoral uptake quantification, where unfortunately there are still unrevealed clues and lack of standardization. We propose an improved method to quantify the biodistribution of gamma-emitting RSA, using tissular corrected uptake indices. We conducted a bi-centric retrospective study on 101 patients with different types of NETs. Three uptake indices obtained after applying new corrections to areas of interest drawn for the tumour and for three reference organs (liver, spleen and lung) were statistically analysed. For the corrected pathological uptake indices, the results showed a significant decrease in the error of estimating the occurrence of errors and an increase in the diagnostic predictive power for NETs, especially in the case of lung-referring corrected index. In conclusion, these results support the importance of corrected uptake indices use in the analysis of ^99m^TcRSA biodistribution for a better personalised diagnostic accuracy of NETs patients.

## 1. Introduction

Neuroendocrine tumours (NETs) originate from the complex hormone-producing neuroendocrine system. This explains both common phenotype features and their great diversity in terms of key structural and functional phenotypic characteristics, molecular profile, localization, aggressiveness, type and site-specific prognosis and response to the treatment [1,2]. Although NETs are sporadic, representing almost 0.66% of all neoplasia [3], they can appear practically ubiquitously in the human body, most frequently in the gastrointestinal tract, lungs and the pancreas [4]. As a consequence of all this diversity, although strong clues in NETs diagnosis exist in terms of biochemical markers [5] and morphological imaging [6], it is not a surprise that a personalised diagnosis is still puzzling and difficulties still can occur during the diagnostic algorithm [7], justifying research to continue to concentrate in this area.

**Figure 1:**
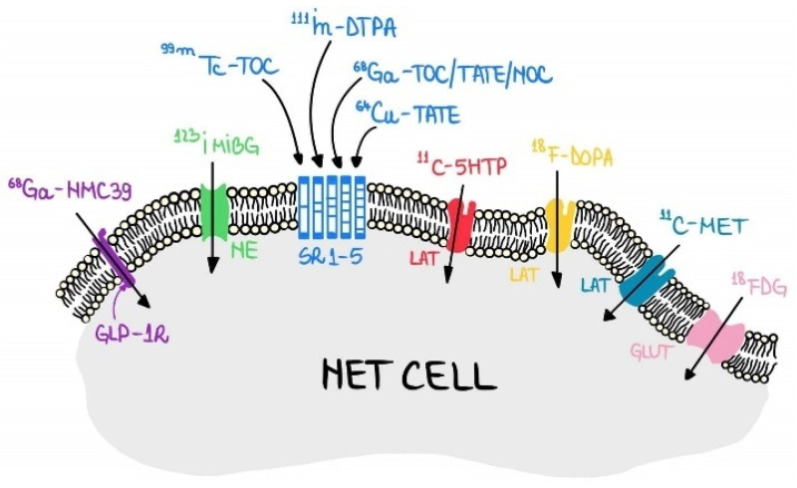
Tracers developed for functional imaging of NETs. Legend: neuroendocrine tumour cell (NET CELL); ^68^Ga-NOTA-MAL-Cys39-exendin-4 (^68^Ga NMC39); glucagon-like peptide-1 receptor (GLP-1R); ^123^I metaiodobenzylguanidine (^123^I MIBG); norepinephrine transporter (NE); ^99m^Tc labelled SA: ^99m^Tc-EDDA-hydrazinonicotinyl-Tyr3-octreotate (^99m^Tc-HYNIC-TATE) and ^99m^Tc-EDDA-hydrazinonicotinyl-Tyr3-octreotide (^99m^Tc-EDDA/HYNIC-TOC; ^99m^Tc-TOC); ^111^In-diethylenetriaminepentaacetic acid-D-Phe1-octreotide (^111^In -DTPA-octreotide); ^68^Ga-labelled somatostatin analogs: ^68^Ga-DOTA-Phe1-Tyr3-Octreotide (DOTATOC), ^68^Ga-DOTA-Nal3 -Octreotide (DOTANOC), and ^68^Ga-DOTA-Tyr3-Octreotate (DOTATATE); somatostatin receptors (SST); ^64^Cu-DOTA-TATE (^64^Cu-TATE); ^11^C-5-hydroxytryptophan (^11^C-5HTP); L-type amino acid transporter (LAT); 6-Fluoro-(^18^F)-l-3,4-dihydroxyphenylalanine (^18^F-DOPA); L-[methyl-^11^C]-methionine (^11^C-MET); ^18^F-2-fluoro-2-deoxy-D-glucose (^18^FDG); glucose transporters (Glut).

In this context, functional NETs imaging emerged at an extremely fast and promising speed, a number of NETs phenotypic markers demonstrating their ability to be visualized with different PET or SPECT tracers (Figure 1). This currently offers a hope for a personalised NETs diagnosis.

Despite their heterogeneity, it is well known that 70% to 100% of NETs share the special mark of somatostatin receptor (SST) overexpression on the cell membrane surface [8,9]. Five SST subtypes are known (SST_1_-SST_5_), with different tumoral proportions, depending on NETs type. For example, in lung NETs: SST_1_—63% to 83%, SST_2_—43% to 96%, SST_3_—5% to 54%, SST_4_—0% to 14%, SST_5_—0% to 71% [10]. GEP-NETs (80%) present all five SST subtypes, in different proportion: SST_1_—68%, SST_2_—86%, SST_3_—46%, SST_4_—93%, SST_5_—57% [11]. The SST distribution in the human body can be accurately visualized by somatostatin receptors scintigraphy (SRS), using radiolabelled somatostatin analogues (RSA). From the five subtypes, the SST_2,5_ have the highest affinity and are the mostly expressed in NETs, being the optimal target for the imaging diagnosis [6,12]. Therefore, SST-based radionuclide imaging, became, currently, a non-invasive, trustworthy and highly sensitive method with a crucial role for the management of these tumours [13,14] and a primary role in the theragnostic approach of NETs [15].

More than one RSA have been introduced along the time, both for SPECT and PET, as is the case of ^111^In -DTPA-octreotide, ^68^Ga-RSA, or ^99m^Tc-RSA, such as ^99m^Tc-EDDA/HYNIC-TOC (^99m^Tc-TOC) [16,17]. Their choice in practice place into balance the method accuracy, the laboratory radiotracer accessibility and the patient option related to the investigation’s cost or radiation exposure.

The accuracy of diagnostic methods is essential for the morphological and functional imaging evaluation in precision oncology. Subtle metabolic changes in functional imaging/pixel-based measurements may indicate a useful early response to therapy that have been found to precede any volume changes of the tumour [18]. The use of various imaging tracers, sometimes in complementarity, may allow a better understanding of the molecular complex phenotype of NETs [19,20]. A careful, standardized, evaluation of the image, both qualitative and quantitative, is essential and required for this final result. Published data demonstrate that there is still a need to develop accurate quantitative parameters in medical image processing, based on understanding of images regarding the cellular uptake mechanism, kinetic and biodistribution of the radiotracer [21,22].

Quantitative functional imaging can contribute to understand of the mechanism of diseases, to assess the condition of the patient-based disease, in order to obtain the best therapeutic effect. Quantitative imaging parameters could be an important tool in personalised medicine, improving patient selection, identifying the population for which treatment would bring the greatest benefits, reducing unnecessary exposure and side effects [21].

Given both the importance of accurate quantification of the tracer uptake and the paucity of systematic recent studies focusing on the field of NETs image quantification, this study aims to contribute to the improvement of the quantification method of the ^99m^Tc RSA uptake for the imaging personalised diagnosis of NETs. We propose a new background correction and quantitative functional uptake indices that can be useful both in diagnostic and theragnostic NETs approach.

## 2. Materials and Methods

### 2.1. Participants

We conducted a retrospective multicentric study, which initially enrolled 107 patients previously diagnosed with NETs. The patients initially included were all patients sent for SRS investigation, over a period of 16 months (from December 2019 to March 2021), in one of the following different nuclear medicine units: Department of Nuclear Medicine, University Emergency Clinical Hospital “St. Spiridon” Iasi, Romania and Department of Nuclear Medicine, Clinical Centre of Kragujevac, Serbia.

Prior to the research program, all approvals were obtained for conducting the study in compliance with the rules of ethics and deontology. When patients presented to the Departments of Nuclear Medicine for SRS, they all signed the informed consent, which includes a section on the use of data for research purposes.

The names of the patients remained anonymous to the study centres.

Patients aged between 18 and 80 years were previously diagnosed with NETs following structural imaging (CT, MRI), neuroendocrine biomarkers assessment and biopsy examinations and were not treated with any SA over the investigation period, the time interval between SRS and the end of biological treatment being at least five weeks. Four patients with incomplete medical records were excluded from the study. Other two patients were excluded because they met certain exclusion criteria: the severe renal and hepatic failure, the inflammatory digestive diseases, (other exclusion criteria being: hypersensitivity to HYNIC-[D-Phe1, Tyr3-Octreotide] trifluoroacetate or to any other excipient, pregnancy and breast-feeding, and the patient’s refusal). 

**Figure 2:**
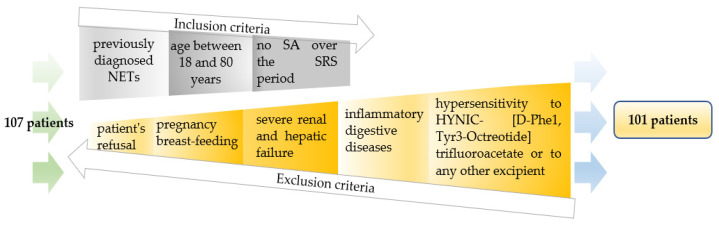
Inclusion and exclusion criteria for patient selection.

Finally, 101 patients for which we performed ^99m^Tc-TOC SRS were enrolled in the study (Figure 2) (51 males, 50 females, and the mean age at the time of diagnosis 55.7 ± 12.1). Two days before investigation, the patients received liquid diet and laxatives on the day preceding ^99m^Tc-TOC SRS to avoid possible false positive results given by the contaminations at the digestive tract level.

### 2.2. Data Acquisition

Each patient received an activity of 10.57 MBq/bw of ^99m^Tc-TOC. A single dose intravenous injection was administered in the cubital vein for each patient and two large-field-of-view gamma-cameras (SIEMENS E.CAM signature series, Dual-Head, Variable Angle, Cardio 2007, Siemens, Medical Systems Inc., Malvern, PA 19355, USA—University Emergency Clinical Hospital “Sf. Spiridon ”Iasi, Romania and SIEMENS E.CAM, Dual-Head, Variable Angle, Syngo 2006, Gold seal, Siemens, Medical Systems Inc., USA—Clinical Centre of Kragujevac, Serbia), fitted with a low-energy, all-purpose, parallel hole collimators and an energy window of 20%, set to 140 keV ± 15% were used. For all patients, early, 2, 4 and 24 h acquisitions were performed. Due to the better target-to-background ratio, the 4 h images have been chosen for quantification, being useful in differentiating normal bowel activity from pathologic lesions, with the muscle uptake and blood activity evidently reduced. 

For whole body (WB) scans acquired at 4 h after tracer’s administration, a 256 × 1024 matrix was used, with zoom of 1 and 8 cm/min bed movement. For image processing/reconstruction parameters we used Syngo software, version Syngo MI Applications VA60C. 

### 2.3. Image and Data Analysis

**Figure 3:**
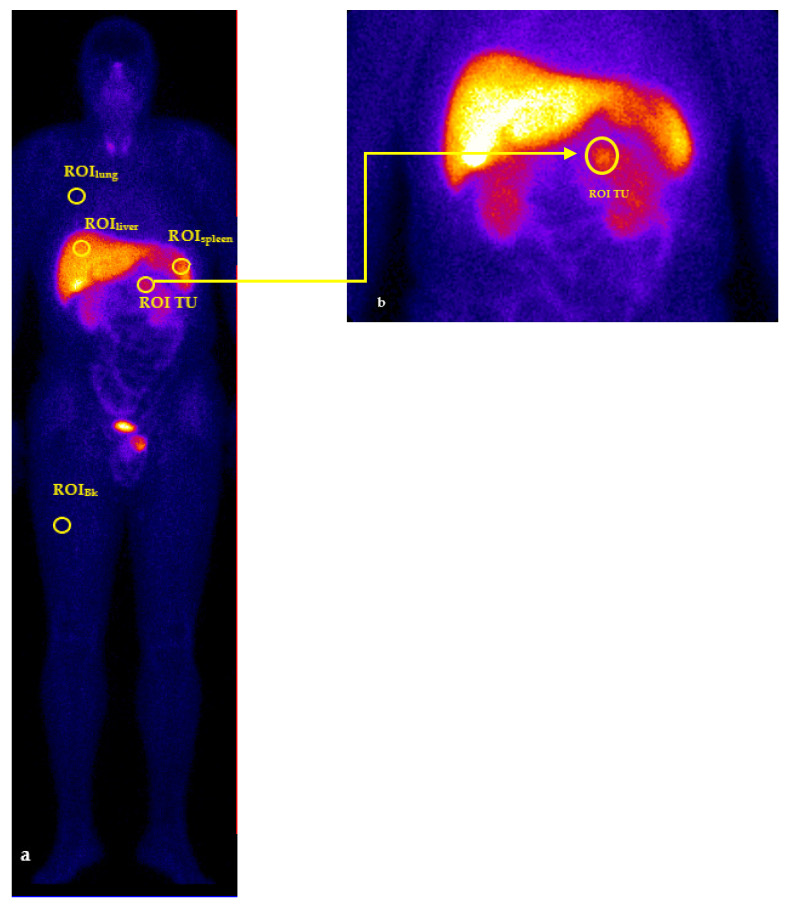
ROI technique. Whole body scintigraphy (**a**): 256 × 1024 matrix, zoom of 1, 8 cm/min bed movement. Static image (**b**): 256 × 256 matrix, zoom of 1. Notes: ROI_TU_: regions of interest for tumour uptake; ROI_liver_: regions of interest for liver; ROI_spleen_: regions of interest for spleen; ROI_lung_: regions of interest for lung; ROI_Bk_: regions of interest for right thigh.

Evaluation of each patient’s study was performed by two nuclear medicine physicians who had over 25 years of experience in interpreting functional imaging studies and working with radiolabelled somatostatin analogues from the beginning of the vector molecule entry on the national market. Any focal tracer accumulation above normal regional tracer uptake was considered as a pathologic finding (tumour uptake). Linear, non-focal limited intestinal uptake was rated as nonspecific, nonpathological uptake. For the quantitative analysis of tumour and main reference organs (liver, spleen and lung) uptake, the data was analysed using a regions of interest (ROI) technique only on anterior views. Identical ROI of 245 pixels were quantified. For each pathological and physiological uptake areas considered to be of study interest and 1/3 upper right thigh was considered to be the reference background region (ROI_Bk_) (Figure 3). For each ROI, total count/pixel ratio was calculated.

We proposed a new background correction (through a correction calculation formula) which implies an activity correction related to the background, as shown in Table 1 that was applied to all the analysed cases.

In order to obtain corrected tumour (which represents in fact pure tumour uptake) or physiological uptake, our background corrections involved subtracting the ROI_Bk_ from the tumour and the chosen reference organ ROIs and dividing the obtained value to the ROI_Bk_ value.

For visualized tumour uptake at the liver level, the value obtained after ROI_liver_ extraction from ROI TU_liver_ was divided to ROI_Bk_, necessary adjustment due to the overlap of intense hepatic uptake.

It is important to use some universal values to quantify uptake (such as our indices), values that are independent of both the geometry of the acquisition and the individual differences between the patients.

The possible variability given by these aspects was the main reason why we tried to find indices that actually analyse the relative quantitative value of pure tracer uptake at the tumour level, a capture that could be independent of the differences between patients.

Three uptake indices were calculated (I_1_ = ROI_tumour_/ROI_liver_, I_2_ = ROI_tumour_/ROI_spleen_, I_3_ = ROI_tumour_/ROI_lung_), and their values were compared before and after the applied corrections, as shown in Table 2.

### 2.4. Statistical Analysis

Statistical data analysis was performed in STATA 16 software (StataCorp LLC, 4905 Lakeway Drive, College Station, Texas 77845-4512, USA) and in SPSS 25 (IBM Corporation, New Orchard Road Armonk, New York 10504-1722, USA). Comparison tests applied to continuous numerical variables were selected based on the distribution of series values and the number of cases included in the analysis. For continuous numerical variables, the Wilcoxon matched pairs test and pair *t* test were applied. The Kolmogorov–Smirnov test (K-S) was applied to verify the normal distribution of the variables. For the series of normally distributed values, the pair *t* test was applied, and for the series that do not respect the normality condition the Wilcoxon matched pairs test was applied. The sets of compared values were pair values, coming from the same patients. However, the homoscedasticity of the series of compared values was tested. For this, the Levene test was applied. The results indicated that there were no significant differences between the variances (*p* > 0.05).

To compare the predictive value of uptake indices calculated according to the corrected vs. uncorrected formula, the receiver operating characteristic (ROC) curve and the AUC value (area under the ROC curve) were evaluated. This analysis was performed both according to the location of neuroendocrine tumours (NETs) but was also applied to the whole group.

The reference value for the significance level (*p*) of the tests applied was considered to be 0.05. A *p* value lower than 0.05 indicated that there is a statistically significant difference.

## 3. Results

The analysed group showed homogeneity in terms of distribution according to the gender of the patients (male: 50.5%, female 49.5%). In addition, the mean age of male patients (54.8 ± 12.8) did not show significant differences (*p* = 0.4401) compared to the mean age of female patients (56.6 ± 11.2).

In the analysed group, the primary gastrointestinal localization of NETs had the highest frequency of 33.7%, followed by MTC with 23.8%. The primary localization at the adrenal glands level had a frequency of only 5.9%. In terms of tumour grading, NETs with unknown grades were in the highest proportion of 36.6%, followed by G2 NETs with 34.7%, the lowest frequency being G3 NETs with 8.9% (Table 3).

We performed the quantitative analysis of the corrected and uncorrected pathological uptake indices (I_1_, I_2_ and I_3_). The corrected and uncorrected pathological uptake indices did not show a normal distribution (Kolmogorov–Smirnov test: *p* < 0.01). Thus, for comparison, the non-parametric test Wilcoxon matched pairs test was applied.

**Figure 4:**
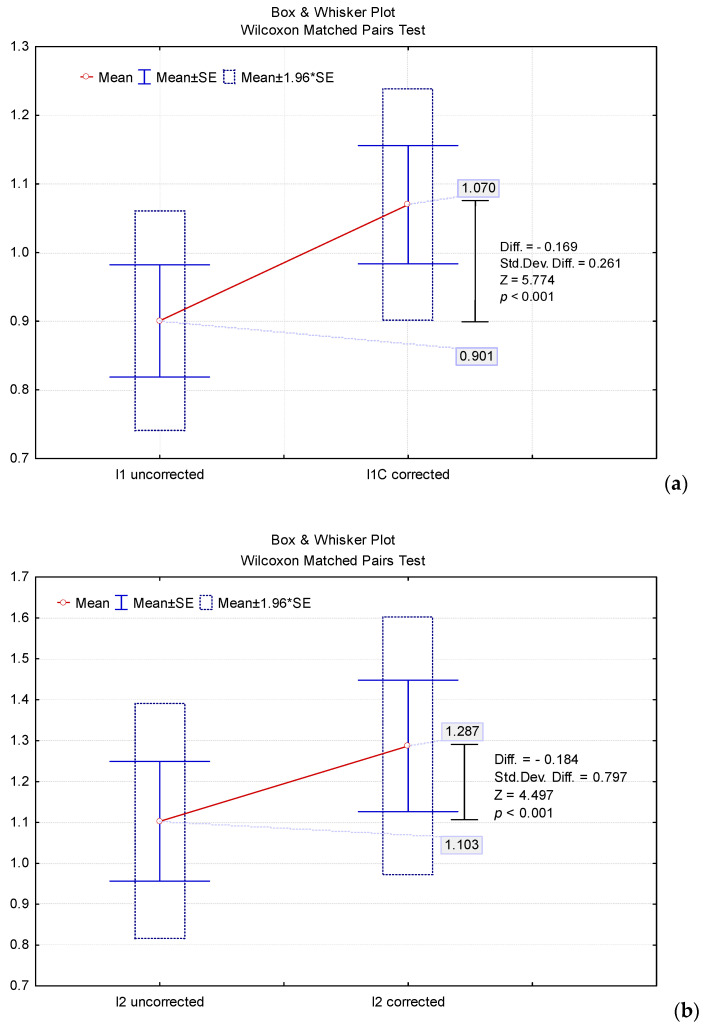

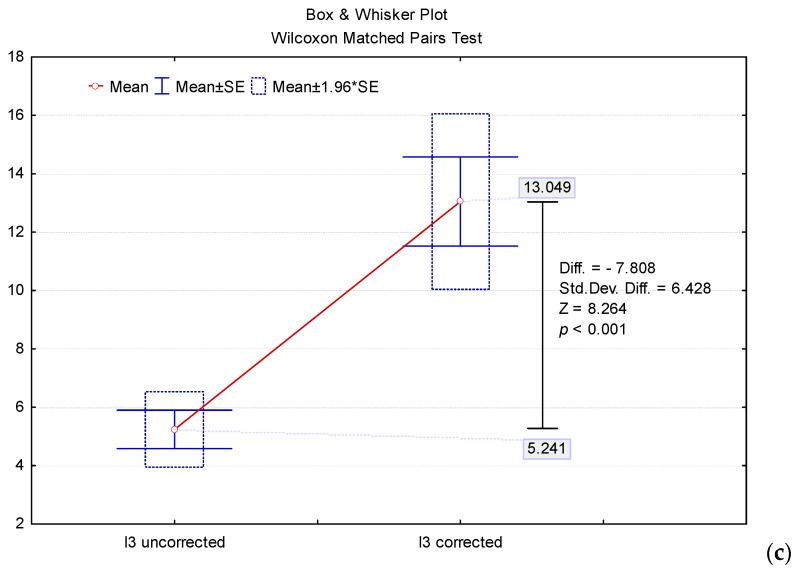
Comparison of uptake indices’ mean values (n = 101): (**a**) I_1_; (**b**) I_2_; (**c**) I_3_.

The analysis of the values of the uptake indices, considering all the patients of the study group (n = 101), showed that the corrected values increased significantly compared to the values obtained based on the reference formula, without any applied correction (Figure 4). The corrected values of the uptake indices were obtained based on the proposed adjustment method for ROI values according to ROI_Bk_ (Table 1).

The significant increase in the values of the I_1c_, I_2c_ and I_3c_ uptake indices can be explained by the background correction (Table 1). If the tumour overlapped other organs (liver), the background correction included subtracting the ROI of that organ from the ROI TU to avoid a possible error given by the summative uptake from the tumour and that organ.

The significant increase of I_3c_ can be explained by the fact that the lung is a good reference due to the lower density of RS, the lung parenchyma presenting only subtypes 1, 2 and 4. Thus, the correct adjustment for the tumoral uptake region (ROI TU) led to identifying a pure tumoral uptake ROI (ROI_C_ TU). 

**Figure 5:**
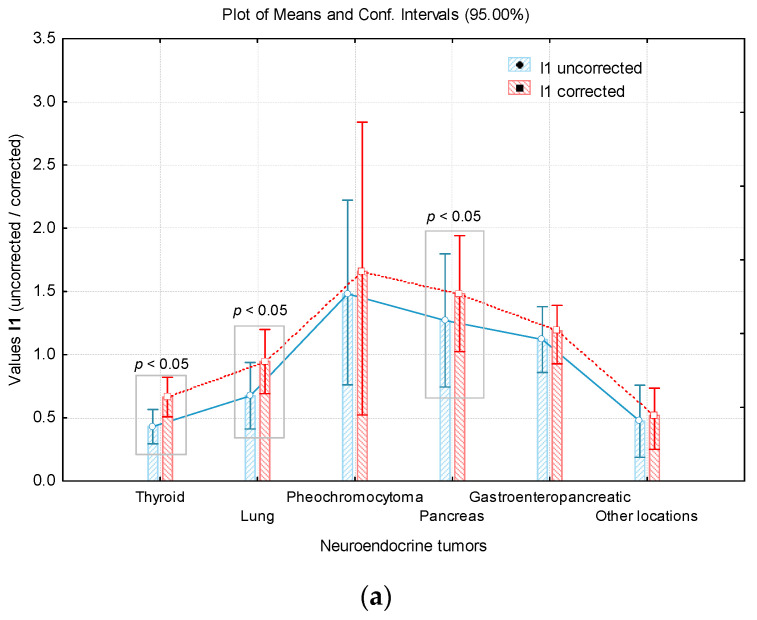

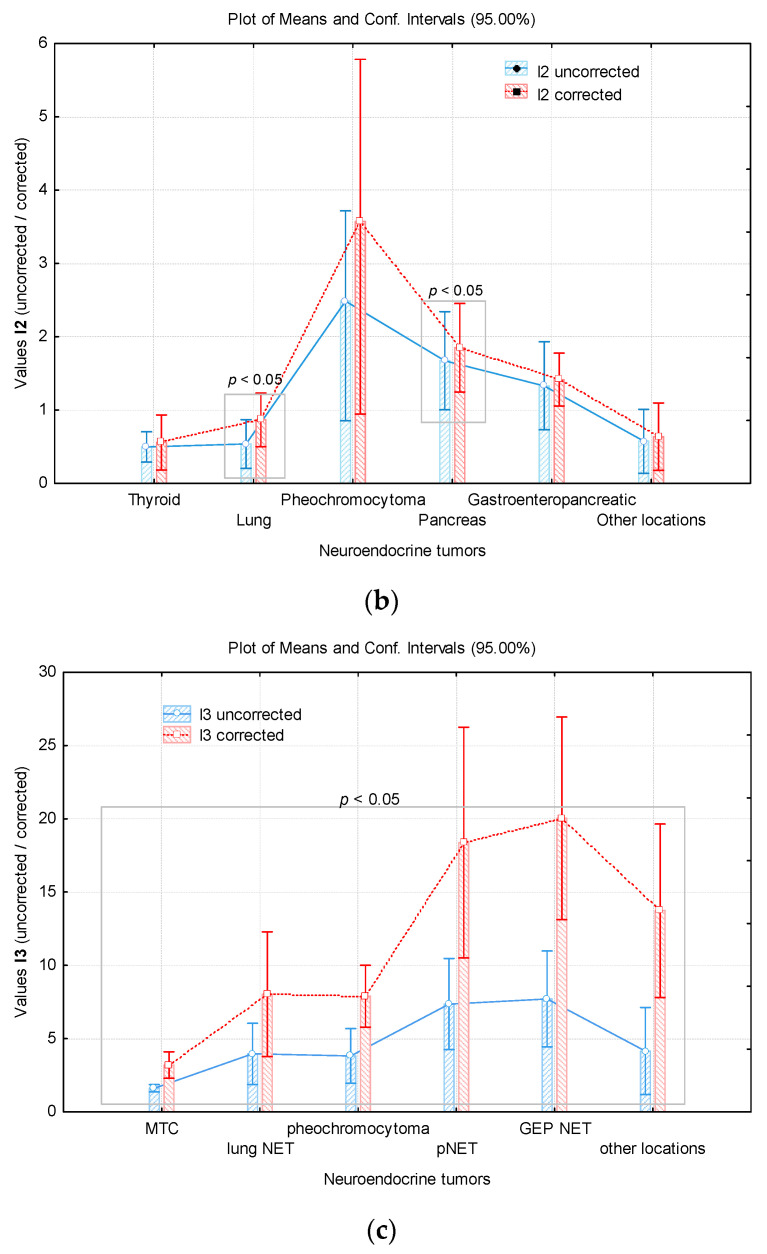
Comparison of mean uptake indices’ values based on primary tumour location: (**a**) I_1_; (**b**) I_2_; (**c**) I_3_.

Depending on the primary NETs location, a comparative study of the values of the uptake indices was performed; to evaluate the differences resulted following the application of the proposed background corrections (Table 4). It is noted that the values increased for all types of NETs included in the study. In the case of the uptake index I_1_, the values obtained by correction are significantly higher (*p* < 0.05) for the primary MTC *(p* < 0.001), lung NETs (*p* = 0.0008) and pNETs (*p* = 0.0069). For the I_2c_ uptake index, significantly higher values were highlighted for the lung NETs (*p* = 0.0002) and pNETs (*p* = 0.0161) (Table 4, Figure 5).

A relevant aspect was noted in the case of the I_3_ uptake index. For all types of primary locations analysed, I_3c_ values were significantly higher compared to uncorrected values (Table 4, Figure 5).

The evaluation of the predictive value for the diagnostic precision power of our quantitative analysis based on the corrected pathological uptake indices was performed based on receiver characteristic curves (ROC).

**Figure 6:**
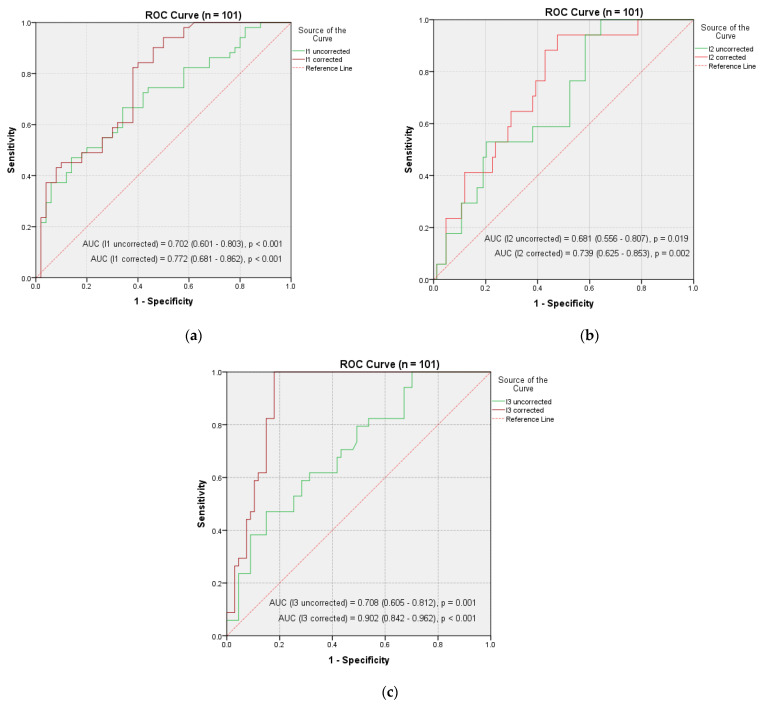
Receptor performance curve (ROC) for comparing the predictive power of uptake indices (I uncorrected vs. I corrected) based on values below the curve (AUC): (**a**) I_1_; (**b**) I_2_; (**c**) I_3_.

The predictive power was assessed considering the area under the curve (AUC) and the calculated estimation error. In the first part of the analysis, the tests were performed for the whole group (n = 101) (Figure 6), followed by a subsequent study of each location of the primary NETs (Table 5).

The use of the values of the corrected pathological uptake indices significantly decreased the error of estimating the occurrence of errors and implicitly increased the predictive power. A significant increase in predictive power was recorded in the case of I_3c_, the AUC value in this case increasing from 0.708 to 0.902 (Figure 6c). These increases were also recorded for I_1c_ and I_2c_, but the amplitude of the increase was smaller (Figure 6a,b). This demonstrates once again that a long-term adjustment increases the accuracy of quantitative assessment of uptake indices for primary NETs locations.

## 4. Discussion

In this study, we quantitatively evaluated the ^99m^Tc-TOC biodistribution on WB SRS scans. The main objective was to improve the quantification approach of the gamma emitter tracer’s uptake in the imaging diagnosis of NETs.

Sheikh introduced the notion of “quantification in theragnostic”, specifying that, although a diagnostic image can qualitatively predict the patient’s response to treatment, for the therapeutic part, the uptake quantification is the one that could be correlated with the level of clinical response [22]. 

Quantifying the pure tumoral uptake of the radiotracer depend on complex tumour/environmental factors such as: the heterogeneity of NETs cellularity (thus tumour cells that may not uptake the tracer at the same time or with the same intensity), the density in various proportions of SST_1-5_ in different cells, the background, the uptakes from other overlapping or organs and tissues, the uptakes ensured by the physiological presence of some SST at those levels or by the vascular tissue reserve. However, the literature does not present extensive analytical studies to evaluate in depth the methods of quantifying the gamma radiation emitting radiotracers biodistribution. 

A number of papers focused on the use of ^99m^Tc RSA SRS in NETs diagnosis. It is evident that all these studies demonstrate the interest for this investigation in NETs diagnosis algorithm, and there is a great diversity on the radiotracer uptake quantification methods, and as a result, these cannot be correctly compared.

There are data which reports that using ^99m^Tc RSA, the SST-overexpressing primary and metastatic NETs lesions can be detected, SRS imaging being more sensitive compared to structural methods (sensitivity of approximately 80% for revealing the site of the primary NETs) [23].

Cwikla and his collaborators compared ^99m^Tc-HYNIC-TOC and ^99m^Tc-HYNIC-TATE uptakes for 12 patients. They calculated target/background ratios using ROIs for the most active areas of tumour, liver, left kidney, with the right lung chosen as background. Their results showed that there was no significant difference regarding the tumour uptake, but only significantly higher liver uptake for ^99m^Tc-HYNIC-TOC [24]. This is different to our results that showed, by applying the proposed correction, that the data with increased precision value are those related to I_3c_, the reference organ for I_3_ being the lung. This difference could be explained by the fact that the summative uptakes were analysed by Cwikla et al., without a ROI_Bk_ subtraction correction.

Briganti, in 2019, published a comparative analysis between ^68^Ga-DOTATOC, ^99m^Tc-HYNIC-TOC and ^111^In-pentetreotide for SST NETs imaging. Their conclusion was that ^99m^Tc-HYNIC-TOC is useful for SST characterization, similar to a good alternative to ^68^Ga-DOTATOC, having a higher imaging quality (the spatial resolution of ^111^In-pentetreotide is 11–14 mm, 7–9 mm for ^99m^Tc-HYNIC-TOC, respectively 4–5 mm for ^68^Ga-DOTATOC) and a lower radiation exposure for patients, compared to ^111^In-pentetreotide [25].

In his semi-quantitative analysis on 10 patients, Decristoforo et al. compared ^111^In-DOTATOC (n = 6), ^111^In-DTPA octreotide (n = 4) and ^99m^Tc EDDA/HYNIC-TOC (n = 10). Tumour/organ ratios were calculated, with ROI plotted for tumour, kidney, liver, spleen, heart and right thigh, on WB scans. The results obtained for ^99m^Tc RSA were superior to ^111^In RSA, with the highest difference for the tumour/kidney and tumour/heart ratios (more than double values in both cases), respectively the lowest for the tumour/liver ratio [23]. Unlike in our study, other reference organs such as kidney and heart were used, but without the adjustment we proposed, correlated with the background activity. The liver and spleen are common reference organs chosen in both studies, but we obtained increased values of uptake indices after we applied the proposed correction.

Gabriel and his collaborators performed a semi-quantitative analysis on 41 patients, using ^99m^Tc-EDDA/HYNIC-TOC and ^111^In-DTPA-octreotide. ROIs for tumour, kidney, liver, spleen, heart and right thigh were plotted. Statistically significant differences (*p* < 0.001) were obtained for tumour/blood, tumour/liver and tumour/kidney ratios for ^99m^Tc-EDDA/HYNIC-TOC compared to ^111^In-DTPA-octreotide uptakes [26]. The study did not involve corrections similar to those proposed by us, although ROI_Bk_ was used.

Hubalewska-Dydejczyk analysed, also semi-quantitatively, 75 patients, using ^99m^Tc-EDDA/HYNIC octreotate, with ROI plotted for each primary and metastatic NETs lesion, important organs (liver, left kidney, spleen) and adjacent normal tissue as background. This paper did not define only one reference ROI_Bk_, the authors analysing the ratios between the target and the adjacent normal tissue. The study showed a high target/non-target ratio, with different values, related to the localization [17].

Comparing ^99m^Tc-EDDA/HYNIC Octreotate with ^111^In-pentetreotide in 14 patients, Deveci et al. obtained, in their semi-quantitative analysis, significantly higher tumour/liver and tumour/kidney ratios and insignificant tumour/spleen and tumour/thigh ratios, showing that ^99m^Tc-EDDA/HYNIC Octreotate is a good imaging method for NETs [27].

In our study of 101 patients with NETs, the choice of quantification on ^99m^Tc-TOC SRS images acquired at 4 h, for all patients, was related to the fact that pathological uptake at this time point was reported to show SST density more precisely than late uptake [28]. The heterogeneity of pathological uptakes, found in all 101 patients included, at different levels, could reflect either the heterogeneity of the tumour, SST density and/or the expression of those five SST subtypes [29].

We proposed the implementation of three uptake indices with correction, in order to improve the accuracy of SRS images interpretation by considering possible error sources. Statistically and comparatively analyse of the diagnostic predictive power of these quantitative uptake indices (corrected vs. uncorrected) sustained the utility of the method. 

Due to the fact that planar images evaluate 2D projections of a 3D scattering activity, the thickness and size of the source can raise issues [30]. Pixel values can be derived from other organs or regions and not from the target region. Thus, the estimated activity will be erroneous at the organ level and the uptake quantification more difficult [31,32]. In addition, tissue vascular intake, in addition to the presence of SST, could be a factor with a major contribution to tracer accumulation, both pathologically and physiologically. Hence, the correction for the overlapping organs and the background activity is highly required and this is the reason we propose the systematically implementation of this correction, to obtain exact values for pure tumour and physiological uptakes, to avoid the summative uptake, in order to reduce the uptake variability evaluation.

Background region localization choice is also important. Different backgrounds were used in different studies, such as liver, lung, bone, heart, thighs and mediastinum, sometimes related to the tumour localisation [23,33]. In our research, we chose only one reference region, the upper third of the right thigh, considered to have a relatively homogeneous SST density, given by tissue vascular supply.

Thus, our quantitative analysis showed that the applied correction brings great benefits for all three uptake indices, regardless of the type of NETs. The obtained results show that for I_3c_, the correction is specific, especially for pNETs, gastrointestinal and adrenal NETs, an index which, uncorrected, has no precision power, as can be seen from the data contained in Table 4. In the case of MTC, although both corrected and uncorrected uptake indices have precision power, the statistical analysis clearly shows that the precision power accuracy for the corrected uptake indices is much increased (*p* < 0.001). This is important in the case of small lesions Krenning 1 NETs, that, usually, do not receive PRRT, because it is considered that tumoral uptake lower than a hepatic one is correlated with a low SST expression. We observed that using background correction it was obtained a better target to non-target ratio and better contrast of images. This possibly means that this kind of NETs patients could be a potential PRRT candidate.

An interesting situation is represented by Krenning 1 MTC lesions, where the uptake is low and the corrected uptake indices show an increased precision diagnosis power.

Regarding the pheochromocytoma, the results obtained have no predictive power, but this aspect may be related to the small number of cases included, which indicates the need for further research on large samples to clarify this issue. However, it must be emphasized that the I_2_ uptake index increases its prediction (AUC I_2_ = 0.642, *p* = 0.245; AUC I_2c_ = 0.728, *p* = 0.031), which suggests visible signs of predictive value improvement. The diagnostic estimation error decreased significantly in the case of I_3c_ and, implicitly, significantly increased the diagnostic accuracy (AUC I_3c_ = 0.768, *p* = 0.027). A higher predictive value is noted when I_3c_ was compared to I_1c_ and I_2c_.

For pNETs I_2c_, the diagnostic estimation error decreases compared to uncorrected I_2_ value. The analysis made for the correction to I_3_ shows a significant increase in the predictive value (AUC I_c_ = 915, *p* < 0.001). The I_1c_ and I_2c_ values promise an increase in predictability and estimation accuracy, which would be more evident in an analysis performed on a larger sample of cases with pNETs. However important, I_3c_ in this context of a small sample size, has a good accuracy. High accuracy and low diagnostic estimation error (*p* < 0.001) support our background corrections. In addition, in the case of gastrointestinal NETs, the quantitative analysis shows an I_3c_ with a high accuracy and a low diagnostic estimation error (*p* < 0.001).

Regarding the dimension of the chosen ROI, we have started from the known fact that the tumour environment is usually cellularly heterogeneous in relation with the malignant cellular markers’ distribution, including SST. As a consequence, the uptake of the radiotracer will be also heterogeneous in the whole tumour. If a ROI that covers the entire tumour will be drawn, cells with a lower number of SST receptors and those with a higher number of receptors will be both included; in this situation, the degree of global radiotracer uptake will quantitatively reflect the totality of the receptors in that tumour. Subsequently, this will be related with the therapeutic tracer uptake in the following PRRT, which will affect all the SST from the tumour by beta radiation. If a small tumoral ROI, which will include only the most intense uptake area of the tumour, will be drawn, the rest of the cells that still express receptors, even if in a smaller number, will be lost from the quantification. This can affect the PRRT monitoring efficiency. This was the reason why the chosen size of ROI was a compromise which covered mostly of tumour sizes of studied patients.

Our analysis shows that the use of uncorrected ROIs is followed by a high probability of errors, their cause being these summative uptakes themselves. The error will impact the diagnostic process of this pathology already known to be extremely heterogeneous, greatly delaying the correct diagnosis. This situation can lead to a late or erroneous use of various therapies, not necessarily in the correct version, with multiple disadvantages for the patient both in the short and long term. 

Image processing with quantitative evaluation is standardized, until now, more for PET imaging, involving the use of standardized uptake value (SUV), metabolic active tumour volume and glycolysis of tumour lesion [34,35]. If standardized PET molecular image processing protocols have already been established [36,37], for SPECT, there is still place to improve. Data from the literature support the oncological concept of differentiation—proliferation which shows that ^18^FDG-PET [38] is useful to show the aggressiveness of NET, while SRS provides information about SST expression [8], the imaging being complementary in relation to Ki67. It is evident that nuclear imaging must continue to develop in parallel for SPECT/PET radiotracers, starting from the complementarity of their cellular uptake mechanisms and balancing risks (cost, ethics, radiation exposure)/benefit for the patient. As known, the irradiation of SPECT/CT scan is less than that of PET/CT, which can represent a major benefit, especially for young ages.

The major limitation of the present study, similar to most published works, is the lack of the histological validation for all patients included. Our results may be of great help in terms of methods, representing the first step for further validation studies. The results are promising for an accurate quantitative assessment of the pathological uptake of gamma-emitting tracers to improve the diagnostic accuracy. They can have great applicability in one of the most important directions of development of imaging methods, artificial intelligence, where quantitative imaging data could successfully support “virtual biopsy” [34], bringing it one step closer to precision medicine and to the ideal goal by which functional imaging would practically achieve an in vivo immunohistochemistry in the field of NETs. The method may be especially useful for response assessment for monitoring of patient evolution under treatment.

## 5. Conclusions

^99m^Tc RSA remain a valuable choice in terms of cost, effectiveness and accuracy of NETs diagnosis. The use of the corrected uptake indices can significantly improve the accuracy of SRS NETs diagnosis, decreasing the variability and possibility of errors, making possible a careful implementation of a precisely targeted personalised therapeutic approach of the patients.

## Figures and Tables

**Table 1:** The ROI correction background correction (a correction calculation formula) proposed.

Corrected activity related to the background	Corrected calculation formula	(ROI TU—ROI_Bk_)/ROI_Bk_ = ROI_c_ TU
	for TU liver localization	(ROI TU_liver_—ROI_liver_)/ROI_Bk_ = ROI_c_ TU
	ROI calculated for normal tissue	(ROI_liver_—ROI_Bk_)/ROI_Bk_ = ROI_c liver_
		(ROI_spleen_—ROI_Bk_)/ROI_Bk_ = ROI_c spleen_
		(ROI_lung_—ROI_Bk_)/ROI_Bk_ = ROI_c lung_

ROI: regions of interest (counts/pixels); ROI TU: regions of interest for tumoral uptake; ROI_Bk_: regions of interest for right thigh; ROIc: corrected regions of interest; ROI_c_ TU: corrected regions of interest for tumoral uptake; ROI TU_liver_: regions of interest for tumoral uptake (liver localization); ROI_liver_: regions of interest for liver; ROI_c liver_: corrected regions of interest for liver; ROI_spleen_: regions of interest for spleen; ROI_c spleen_: corrected regions of interest for spleen; ROI_lung_: regions of interest for lung; ROI_c lung_: corrected regions of interest for lung.

**Table 2:** Calculation formulas for uncorrected and corrected uptake indices.

Indices	Uncorrected Indices	Corrected Indices
I_1_	I_1unc_ = ROI TU/ROI_liver_	I_1c_ = ROI_C_ TU/ROI_c liver_
I_2_	I_2unc_ = ROI TU/ROI_spleen_	I_2c_ = ROI_C_ TU/ROI_c spleen_
I_3_	I_3unc_ = ROI TU/ROI_lung_	I_3c_ = ROI_C_ TU/ROI_c lung_

I_1_: uptake index 1; I_2_: uptake index 2; I_3_: uptake index 3; unc: uncorrected; c: corrected; ROI TU: regions of interest for tumoral uptake; ROI_liver_: regions of interest for liver; ROI_c liver_: corrected regions of interest for liver; ROI_spleen_: regions of interest for spleen; ROI_c spleen_: corrected regions of interest for spleen; ROI_lung_: regions of interest for lung; ROI_c lung_: corrected regions of interest for lung.

**Table 3:** Descriptive characteristics for the patients included in the study.

Baseline Characteristics	Patients with Neuroendocrine Tumours (*n* = 101)	
Age: years, mean ± SD	55.7 ± 12.1	Std. Err.: 1.2
Gender, (male/female), *n* (%)	51/50 (50.5%/49.5%)	
Neuroendocrine tumours (NETs) localization, *n* (%) thyroid lung pheochromocytoma pancreas gastroenteropancreatic other locations	24 (23.8) 13 (12.9) 6 (5.9) 17 (16.8) 34 (33.7) 7 (6.9)	−
Grade, *n* (%) G1 G2 G3 Unknown	20 (19.8) 35 (34.7) 9 (8.9) 37 (36.6)	−
Ki67%, mean ± SD	13.4 ± 13.8	Std. Err.: 1.8
Metastasis (Yes/No), *n* (%)	65/36 (64.4/35.6)	

SD, standard deviation; Std. Err., standard error.

**Table 4:** Uncorrected and corrected uptake indices values depending on the localization of the neuroendocrine tumour.

Neuroendocrine Tumours(localization)	I Uncorrected	I Corrected	
	I1 = ROI TU/ROI Liver(Mean ± Standard Deviation)		
	I_1_	I_1c_	*p*‒Value ^§^
Thyroid	0.43 ± 0.32	0.66 ± 0.37	<0.001 *
Lung	0.67 ± 0.43	0.95 ± 0.42	0.0008 *
Pheochromocytoma	1.48 ± 0.65	1.66 ± 0.08	0.3483
Pancreas	1.27 ± 1.03	1.48 ± 0.79	0.0069 *
Gastrointestinal	1.12 ± 0.74	1.19 ± 0.67	0.0944
Other locations	0.47 ± 0.27	0.52 ± 0.37	0.2193
	I1 = ROI TU/ROI spleen(mean ± standard deviation)		
	**I_2_**	**I_2c_**	
Thyroid	0.49 ± 0.31	0.56 ± 0.39	0.4799
Lung	0.53 ± 0.24	0.87 ± 0.41	0.0002 *
Pheochromocytoma	2.48 ± 1.08	3.57 ± 1.96	0.2205
Pancreas	1.67 ± 0.93	1.85 ± 0.92	0.0161 *
Gastrointestinal	1.32 ± 0.72	1.41 ± 0.34	0.6251
Other locations	0.57 ± 0.27	0.64 ± 0.29	0.1027
	I1 = ROI TU/ROI lung(mean ± standard deviation)		
	**I_3_**	**I_3c_**	
Thyroid	1.61 ± 0.59	3.19 ± 0.97	<0.001 *
Lung	3.95 ± 1.45	8.03 ± 2.03	0.0440 *
Pheochromocytoma	3.81 ± 0.78	7.90 ± 2.01	0.0016 *
Pancreas	7.35 ± 1.03	18.38 ± 6.32	0.0022 *
Gastrointestinal	7.71 ± 3.37	20.03 ± 8.81	<0.0001 *
Other locations	4.14 ± 1.21	13.73 ± 4.96	0.0364 *

^§^ Pair *t* test; * Marked effects are significant at *p* < 0.05.

**Table 5:** Uptake indices evaluated for their diagnostic value.

NETs (Localization)	Uptake Indices	Area under the Curve AUC (95%CI)	Std. Error	*p*-Value
Thyroid	I1 uncorrected	0.714 (0.606–0.823)	0.055	0.002 *
	I1 corrected	0.808 (0.707–0.909)	0.052	<0.001 *
	I2 uncorrected	0.723 (0.605–0.841)	0.060	0.001 *
	I2 corrected	0.817 (0.695–0.938)	0.062	<0.001 *
	I3 uncorrected	0.75 (0.674–0.926)	0.039	0.002 *
	I3 corrected	0.887 (0.822–0.953)	0.034	<0.001 *
Lung	I1 uncorrected	0.632 (0.774–0.926)	0.039	0.043 *
	I1 corrected	0.829 (0.822–0.953)	0.034	<0.001 *
	I2 uncorrected	0.663 (0.419–0.707)	0.073	0.032 *
	I2 corrected	0.776 (0.505–0.848)	0.088	0.041 *
	I3 uncorrected	0.692 (0.371–0.731)	0.092	0.003 *
	I3 corrected	0.891 (0.413–0.769)	0.091	0.001 *
Pheochromocytoma	I1 uncorrected	0.496 (0.21–0.783)	0.046	0.977
	I1 corrected	0.614 (0.398–0.83)	0.010	0.350
	I2 uncorrected	0.642 (0.401–0.883)	0.023	0.245
	I2 corrected	0.728 (0.694–0.892)	0.084	0.031 *
	I3 uncorrected	0.653 (0.324–0.736)	0.054	0.074
	I3 corrected	0.768 (0.435–0.702)	0.068	0.027 *
Pancreas	I1 uncorrected	0.631 (0.556–0.807)	0.064	0.079
	I1 corrected	0.689 (0.625–0.853)	0.058	0.062
	I2 uncorrected	0.645 (0.61–0.859)	0.063	0.062
	I2 corrected	0.696 (0.647–0.853)	0.052	0.051
	I3 uncorrected	0.794 (0.576–0.812)	0.060	0.012 *
	I3 corrected	0.915 (0.875–0.986)	0.051	<0.001 *
Gastrointestinal	I1 uncorrected	0.576 (0.355–0.798)	0.062	0.211
	I1 corrected	0.689 (0.486–0.891)	0.052	0.072
	I2 uncorrected	0.599 (0.488–0.711)	0.057	0.104
	I2 corrected	0.642 (0.336–0.749)	0.054	0.080
	I3 uncorrected	0.672 (0.567–0.757)	0.054	0.041 *
	I3 corrected	0.908 (0.785–0.982)	0.053	<0.001 *
Other locations	I1 uncorrected	0.579 (0.49–0.868)	0.096	0.115
	I1 corrected	0.59 (0.448–0.893)	0.088	0.053
	I2 uncorrected	0.519 (0.416–0.821)	0.103	0.297
	I2 corrected	0.56 (0.467–0.853)	0.098	0.160
	I3 uncorrected	0.706 (0.279–0.772)	0.036	0.028 *
	I3 corrected	0.872 (0.794–0.98)	0.029	0.001 *

Std. Error, standard error; CI, confidence interval; * marked effects are significant at *p* < 0.05.

## Data Availability

The data presented in this study are available on request from the corresponding author.
